# Cloud Health Resource Sharing Based on Consensus-Oriented Blockchain Technology: Case Study on a Breast Tumor Diagnosis Service

**DOI:** 10.2196/13767

**Published:** 2019-07-23

**Authors:** Xiaobao Zhu, Jing Shi, Cuiyuan Lu

**Affiliations:** 1 School of Software Nanchang Hangkong University Nanchang China; 2 Department of Mechanical and Materials Engineering University of Cincinnati Cincinnati, OH United States

**Keywords:** blockchain, cloud health, breast tumor diagnosis, k-nearest neighbors (KNN), Proof of Authority (PoA), consensus-oriented

## Abstract

**Background:**

In recent years, researchers have made significant efforts in advancing blockchain technology. This technology, with distinct features of decentralization and security, can be applied to many fields. In areas of health data and resource sharing, applications of blockchain technology are also emerging.

**Objective:**

In this study, we propose a cloud health resource-sharing model based on consensus-oriented blockchain technology and have developed a simulation study on breast tumor diagnosis.

**Methods:**

The proposed platform is built on a consortium or federated blockchain that possesses features of both centralization and decentralization. The consensus mechanisms generate operating standards for the proposed model. Open source Ethereum code is employed to provide the blockchain environment. Proof of Authority is selected as the consensus algorithm of block generation.

**Results:**

Based on the proposed model, a simulation case study for breast tumor classification is constructed. The simulation includes 9893 service requests from 100 users; 22 service providers are equipped with 22 different classification methods. Each request is fulfilled by a service provider recommended by the weighted k-nearest neighbors (KNN) algorithm. The majority of service requests are handled by 9 providers, and provider service evaluation scores tend to stabilize. Also, user priority on KNN weights significantly affects the system operation outcome.

**Conclusions:**

The proposed model is feasible based on the simulation case study for the cloud service of breast tumor diagnosis and has the potential to be applied to other applications.

## Introduction

### Background

Health care is closely related to the survival and happiness of human beings, and thus the efficiency and effectiveness of health care are of critical importance. The health care industry is one of the most important industries for developed and developing countries. According to a report from the World Health Organization, in 2018 total health care expenditure grew by 4% for high-income countries, while this figure reached approximately 6% for low- and middle-income countries. In either group of countries, the growth rate of health care expenditure is higher than that of gross domestic product [1].

In recent decades, information and computer technologies have significantly improved the efficiency of health care delivery and access to care and greatly reduced the waste of health care resources. Besides the well-known examples of electronic health records (EHRs), telemedicine, and clinical decision support systems, applications of new technologies such as mobile health and artificial intelligence (AI) are booming. For instance, HealthTap [2], a popular health app, offers health services at no additional charge to policyholders that include asking a network of licensed physicians health-related questions online by connecting immediately or by appointment with a doctor for consultation via video conference, phone call, or text chat. It has now attracted more than 140,000 licensed doctors in good standing from 170 countries. Also, Google’s DeepMind Health [3] is a health AI system collaborating with the National Health Service in the United Kingdom. The goal is to deliver better care for millions of people worldwide.

However, a rising number of issues have been reported along with the digitalization and informatization in health care fields. It is well recognized that information systems can fail to deliver the best solution for the patients due to the lack of necessary information [4]. Adler-Milstein et al [5] illustrated that customized and incompatible health systems can cause gaps in communication and coordination between medical organizations. Zhang et al [6] believed that in health information systems, one fundamental problem is the lack of a trusted platform that can connect independent health systems and provide an end-to-end reachable network. Similarly, Zhang et al [7] also indicated that pressing issues in the health field include fragmented and siloed data, delayed communications, disparate workflow tools, and the lack of a health care resource–sharing platform. In this regard, blockchain technology, with its unique characteristics such as decentralization, consensus, cryptocurrency, and immutability of data, provides a novel tool to address these issues.

In this study, we propose a cloud health resource–sharing model based on consensus-oriented blockchain technology and illustrate the model with a case study on breast tumor diagnosis.

### Literature Review

#### Blockchain in Health Data Sharing

Health data sharing has been one of the biggest challenges for health care organizations. Since Bitcoin was first introduced in 2008 by the pseudonymous creator Satoshi Nakamoto [8], it has experienced amazing development. Blockchain, which is the core technology of Bitcoin, has drawn unprecedented interest and attention. In the past several years, researchers and practitioners have started to recognize the value of blockchain technology for addressing data sharing challenges. Rifi et al [9] illustrated the specific problems such as privacy, scalability, and interoperability and highlighted the benefits of blockchain technology in the deployment of a secure and scalable solution for medical data exchange. Xia et al [10] addressed patient privacy risks of disseminating medical data beyond the protected cloud of institutions and proposed a blockchain-based data-sharing framework. The framework addresses access control challenges associated with sensitive data stored in the cloud using the immutability and built-in autonomy properties of blockchains. Liang et al [11] proposed an innovative user-centric health data-sharing solution by using a decentralized and permissioned blockchain for privacy protection and identity management improvement. More recently, Alexaki et al [12] also considered blockchain technology for supporting the decentralized care cycle. With blockchain technology, patient privacy and medical record integrity is addressed, while efficient interoperability between providers is simultaneously ensured. Zhang et al [5] illustrated the contributions of blockchain technology for clinical data sharing in the context of technical requirements defined in the Shared Nationwide Interoperability Roadmap from the Office of the National Coordinator for Health Information Technology. In addition, Ji et al [13] proposed a location-sharing scheme for telecare medical information systems by using blockchain technology. In their work, basic requirements of the scheme such as decentralization, confidentiality, and verifiability were defined and an experiment was conducted to demonstrate the efficiency and feasibility of the proposed scheme.

#### Blockchain in Electronic Health Records

In literature, numerous studies have applied blockchain technology to EHR management. Dey et al [14] developed a solution of reliable storage of health data by proposing a blockchain–Internet of Things model where a biosensor measures and collects real-time data concerning a patient’s medical status and stores the data in the blockchain. The InterPlanetary File System protocol was proposed to save discharged patient records, thus reducing the load on the actual blockchain. Li et al [15] proposed a novel blockchain-based data preservation system for medical data in which users can preserve the essential data and the originality of data. A prototype of the data preservation system was implemented based on the real-world blockchain-based platform Ethereum, and the results demonstrated the effectiveness and efficiency of the proposed system. Dagher et al [16] proposed a blockchain-based framework called Ancile. The Ancile framework can provide secure, interoperable, and efficient access to medical records for patients, providers, and third parties while preserving the privacy of patient-sensitive information. Chen et al [17] designed a storage scheme to manage personal medical data based on blockchains and cloud storage. Furthermore, a service framework for sharing medical records was described. Also, the characteristics of the medical blockchain were presented and analyzed through comparison with traditional systems. Wang and Song [18] proposed a secure EHR system with an attribute-based cryptosystem for medical data, identity-based encryption for digital signatures, and blockchain technology for the integrity and traceability of medical data. Similarly, Guo et al [19] indicated there is a critical need for patients to pay close attention to their own health care information and medical data storage. An attribute-based signature scheme with multiple permissions was proposed to ensure the effectiveness of EHR infused in the blockchain.

Zhang et al [20] described the issues of system evolvability, storage requirements minimization, patient data privacy protection, and application scalability across a large number of users. These challenges can be mitigated in a blockchain-based decentralized application (DApp) for smart health. Brogan et al [21] discussed how distributed ledger technologies can play a key role in advancing electronic health by ensuring the authenticity and integrity of data generated by wearable and embedded devices. Tian et al [22] proposed to establish a shared key that could be reconstructed by the legitimate parties before the process of diagnosis and treatment begins. The data in the diagnosis and treatment process are encrypted and stored in a blockchain using the shared key.

#### Blockchain in Medicine Prescription Tracking

Blockchain technology provides the health industry a new vision for drug tracking, in particular opioid prescription tracking. Mettler et al [23] demonstrated the examples of public health care management, user-oriented medical research, and drug counterfeiting in the pharmaceutical sector. The examples were believed to be just the starting points for blockchain technology to be adopted in the health care industry. Dhillon et al [24] proposed a blockchain system in which a provider can check the blockchain to find a currently active prescription when writing a prescription. An active prescription from a different provider will automatically invalidate the request for a new prescription, and this can be encoded as a second spending request in the network. Meanwhile, efforts have been made by major blockchain participants such as IBM and Deloitte blockchain laboratories to control opioid overdose epidemics. Zhang et al [5] indicated that blockchain-based systems can build a trusted network of hospitals and pharmacies to store drug-related transactions in a responsible way. The distributed and shared-licensed blockchain platform allows loosely coupled providers to access other data silos without a clear trust relationship among them. Taylor and Hare [25] employed the permissions and restrictions associated with the digital wallet to interact with unexpected events and requirements of transactions contained in blocks. This interaction can be used to realize the verification of opioid dose ownership. It may also include provisions for the sale of opioids that involve current owners, patients, and drug abuse agencies.

#### Blockchain in Clinical Trials and Precision Medicine

The online service of clinical trials and precision medicine is becoming increasingly popular. Blockchain technology provides a trustworthy safety mechanism for this service. Shae and Tsai [26] proposed a blockchain platform for clinical trials and precision medicine, and they identified four new system architecture components required to be developed on top of the traditional blockchain. Suzuki et al [27] proposed a scheme to record both client requests and server replies in an auditable manner using blockchain technology as a request-response channel for a client-server system. A proof-of-concept algorithm was developed based on a publicly available blockchain testbed. Tsai [28] proposed a mechanism for transforming repetitive blockchain calculations into a distributed parallel computing architecture. In the process, smart contracts are adopted to support mobile computing. The mechanism is elucidated to establish real-world evidence of clinical trials for individuals and precision medicine. Benchoufi et al [29] adopted blockchain technology to build a consent workflow. The proposed proof-of-concept protocol includes the use of a blockchain to time-stamp each step in the patient’s consent to collect clinical trial information in a secure, indivisible, and transparent manner through cryptographic verification. A single document is obtained in an open format that explains the entire consent collection process. It is believed that in the future, blockchains can be used to track complex data from clinical trials, and streaming smart contracts can help prevent clinical trial events from occurring in incorrect chronological order.

## Methods

### Concept of the Blockchain-Based Cloud Health Service-Sharing Model

The proposed system presents a new model of consensus-oriented health data and service sharing by integrating the blockchain technologies with the concept of cloud computing. Blockchain techniques such as public key and private key technology, virtual currency, smart contract, consensus algorithm, and HASH256 encrypted technology are used for automatic consensus-driven services and sharing of value. The services allow health care organizations, health platforms, individuals, and health-relevant industry to share the increasing system value and possess a variety of safe, reliable, credible, high-quality, inexpensive, easily payable, and on-demand health resources.

The proposed health service–sharing model is based on an open source system. All of the consensus standards and system recommendation algorithms are open source to the approved users. The proposed model adopts the consortium or federated blockchain structure [30]. Therefore, the blockchain system in the proposed model is not fully decentralized. Instead, the consensus-oriented centralized model helps the system to stay away from the potential issues of a fully decentralized system such as crime and volatility. Users in the proposed model are divided into three major categories: administrators, service providers, and regular users. Administrators typically include signers and members of arbitration committees. The information of service providers is pre-verified by an administrator, and the administrators vote through smart contracts. The blockchain system only stores transaction and summary information related to system components such as the basic machine specification. However, there are many types of information (eg, medical images) that need to be verified by the blockchain system in the proposed model. Those data are typically saved in a distributed storage system and verified by the Oracle mechanism. Oracle in a blockchain system provides trusted entities that allow the blockchain system to access external data [31]. The Oracle mechanism also guarantees the safety of external data blockchain data.

Value sharing is the core concept of the proposed model. The model employs a cryptocurrency system, the cloud health coin system, and the cryptocurrency is called cloud health coin (CHC). In the blockchain system of the proposed model, only signers can mine blocks. Therefore, Proof of Authority (PoA) [32] is employed as the network consensus algorithm rather than Proof of Work. Moreover, the practical Byzantine fault tolerance protocol can ensure system safety even with individual signer errors. With the PoA consensus algorithm, it is not necessary for signers to invest significant funds for computing power competition. Besides the conventional design in which signers are entitled to all the mining rewards, we provide another option in which a considerable proportion of mining rewards is transferred into a system public account. The public fund is used to support a standard verification smart contract, which rewards data sharing and those who have contributed to the system. For instance, the equipment that has spare computing power and is involved in the distributed computing of the recommending provider will receive a reward.

### Hierarchical Structure for Everything as a Service on Proposed Model

**Figure 1:**
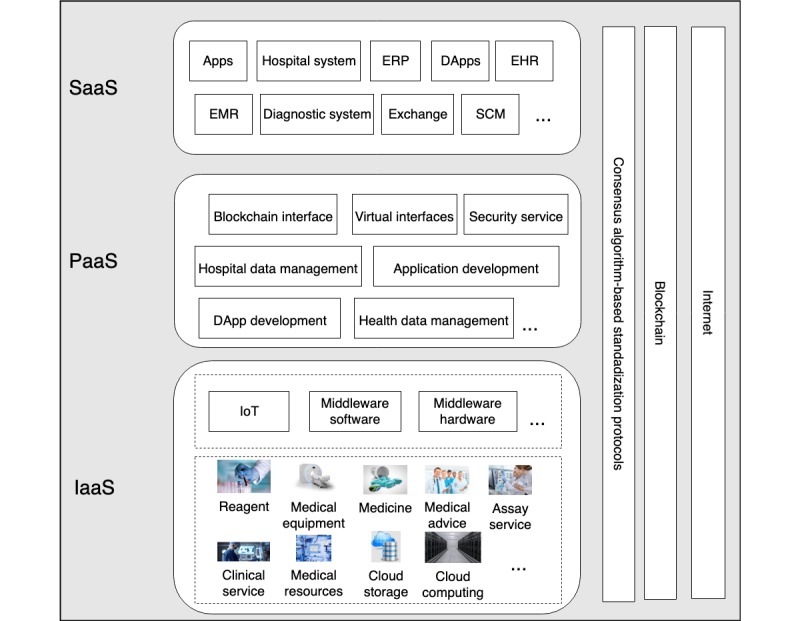
Hierarchical structure for everything as a service for the proposed model. SaaS: software as a service; PaaS: platform as a service; IaaS: infrastructure as a service; ERP: enterprise resource planning; DApp: decentralized application; EHR: electronic health record; EMR: electronic medical record; SCM: supply chain management; IoT: internet of things.

The concept of *everything as a service* is adopted from cloud computing and applied in the proposed model. Everything is virtualized to serve customers by applying standards of consensus mechanism through the smart contracts. Also, the system allows the health platform to use and share the health data comfortably and conveniently. Physical cloud health resources deliver *infrastructure as a service* by core middleware capabilities. The user level middleware and system developer tools provide *platform as a service* capabilities. The various application services, DApps, and professional software offer *software as a service* capabilities. Figure 1 shows the hierarchical structure for *everything as a service* on the proposed model.

The *software as a service* layer provides the services of many software applications to the end users. The software can be provided by the proposed system or third-party companies or even developed by users. The majority of the software packages are not open source. Examples of software applications include hospital management systems, EHRs, electronic medical records, and CHC exchange. The *platform as a service* layer offers software frameworks that help developers create apps, DApps, or other *software as a service* layer software. This level also provides a number of open source tools preapproved by smart contracts. These tools allow developers to develop apps or DApps according to relevant system standards comfortably. All health care organizations provide the interface of standardized data access (with consensus). Middleware software enables the data from various platforms to have a standard format. The *platform as a service* layer also includes service level agreements, accounting, billing, Oracle, and blockchain system interfaces.

The *infrastructure as a service* layer includes medical systems for diagnosis and treatment, laboratory equipment, computers, and other health resources that can be made available on the cloud. In the proposed model, the service providers offer services according to consensus standards. The middleware helps service providers deliver services through a virtualization process.

### Case Study on Cloud-Based Breast Tumor Diagnosis

Assume that a breast cancer patient who lives in a developing country or rural area needs laboratory examination of her biological sample to identify the cancer type. Hospitals in her country or area cannot identify the biological sample, so the patient must resort to hospitals or laboratories in other countries. The challenges now: How can the patient find an appropriate organization for the diagnosis in another country? How can the patient schedule services from the identified organization? How can the patient pay for the services?

**Figure 2:**
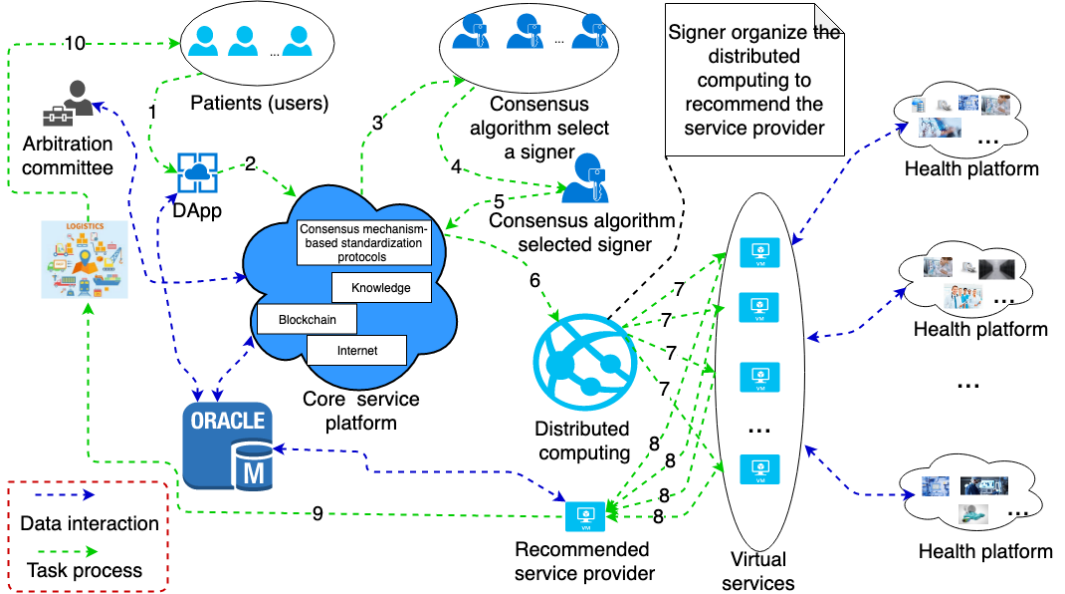
Breast tumor diagnosis based on the proposed model. DApp: decentralized application.

With the proposed model, those challenges faced by the patient can be addressed. First, all service providers in the system are preapproved. The system uses open source algorithms to recommend providers who are qualified. The nature of open source algorithms ensures impartial and more credible recommendation results compared with the results of an internet search using commercial search engines. Virtual currency is applied to pay for the transaction, which frees the patient from the traditional currency exchange. The patient can simply submit a service request, often with an affordable cost limit, to the system and wait for the notification. As shown in Figure 2, the system responds to a service request initiated by the patient (user). The system will provide the estimated cost range based on a big data analysis of past similar services and can provide a convenient service to buy CHC. Meanwhile, the system will select a signer to organize the computing process for recommending a service provider according to consensus mechanism. If the concept of a public account is adopted, a number of users and providers can be involved in distributed computing and obtain their computing rewards. This can enable users and providers who are not signers to share the system value by dividing up the gas and mining rewards with the signer. The providers related to this service request receive the detailed job requirement and payment information. All process data are written onto the blockchain for retrospective purposes.

Thereafter, patients/users will have the test sample prepared according to the system standard. When the test sample is ready, the processes will start and all related processes are monitored under open source algorithms. After each process is finished, the patient can rate the services. The result of the evaluation will be written to the blockchain. If a provider does not agree with the assessment by the patient, an application of arbitration can be submitted. All shreds of evidence guarantee authenticity and credibility of the testimonies. During the process, a vote will happen in the arbitration committee. This vote relies on data reports and is realized by the smart contract.

**Figure 3:**
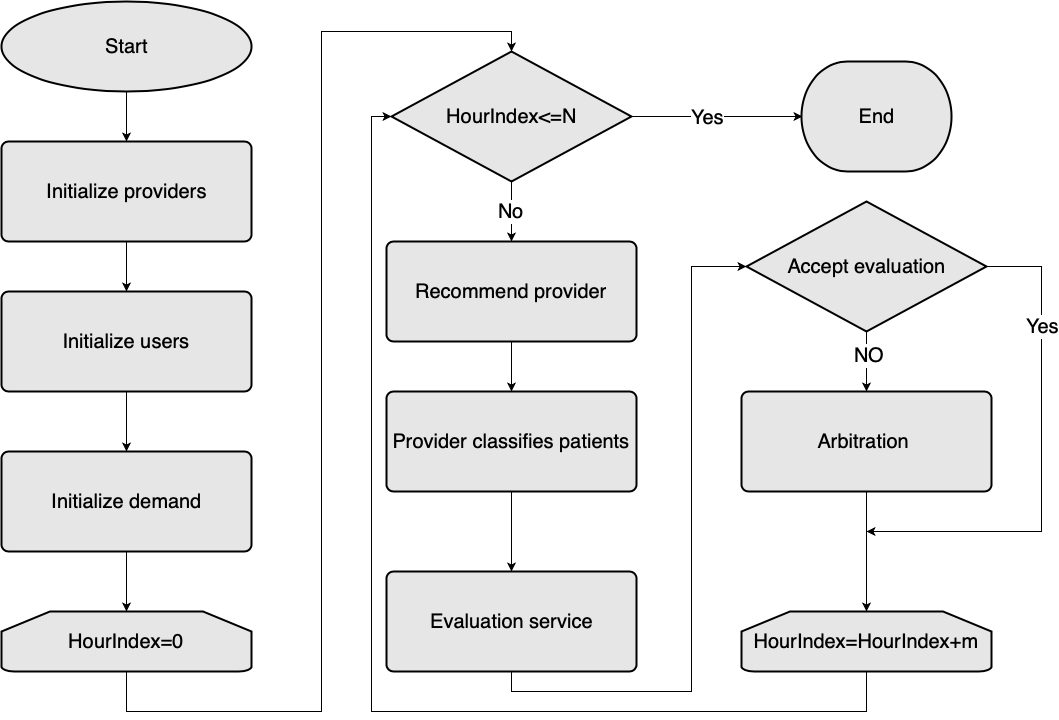
Simulation flowchart.

### Simulation

We implemented the proposed model based on the open source blockchain system Ethereum. The simulation was coded in Python 3.6 with a Spyder integrated development environment carried out on a Mac system with a 2.2 GHz Intel i7 CPU and 16 GB DDR4 memory. Three computers running macOS, Ubuntu, and Windows operating systems were employed to simulate the cross-platform scenario with multiple operating systems. We constructed a consortium or federated blockchain based on Ethereum. The system assumes 9 predetermined signers (also can be called owners), who are the only accounts that can mine the blocks according to the PoA consensus algorithm, and 22 providers who can provide the breast cancer diagnosis service. One hundred users are created to make service requests. A Poisson distributed random function is used to simulate the interarrival time of job requests. The purpose of the simulation is to gain insights into the performance and evaluation of providers. Figure 3 shows the simulation flowchart, which includes initiation, provider recommendation, and service evaluation.

### Provider Estimation

Based on the Wisconsin Diagnosis Breast Cancer Database [33,34], researchers have developed methods for breast cancer data classification. Table 1 summarizes the methods and their accuracies of classification according to the literature. In this study, each method is treated as a provider.

Considering smart contracts have preapproved all providers, the provider’s initial service evaluation score is set to be 6 out of 10. In the 0 to 10 evaluation scale, 0 represents the worst, while 10 represents the best. A patient document is regarded as a data unit. Providers have different pricing strategies on the unit price. Here, we assume that a higher accuracy requirement leads to a more expensive diagnosis cost. The unit price (*p*_unit_) is calculated by *p*_unit_= *δ* + *ε* * *f* (*α*), in which *δ* and *ε* are constants, *δ* represents the base price, *ε* represents the price variance caused by the accuracy rate of diagnosis, and *f* (*α*) is a normal distribution random-based function that ranges from 0 to 1. The mean of *f* (*α*) equals *α*, which is the normalized diagnostic accuracy.

**Figure 4:**
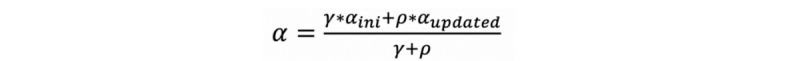
Equation of computing dynamic diagnostic accuracy.

**Figure 5:**
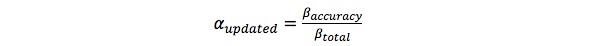
Equation of computing historical dynamic diagnostic accuracy.

The diagnostic accuracy has a stochastic nature associated with the number of jobs serviced. The initial diagnosis is defined by Table 1. The simulation has a computer diagnosis function. The diagnostic accuracy of each provider will change as the number of jobs fulfilled increases. The dynamic diagnostic accuracy is calculated as seen in Figure 4, in which *γ* is a constant to prevent an accidental diagnosis that affects the provider’s accuracy too much. In this case, *γ* equals 1000, *α*_ini_ is the initial diagnostic accuracy of a provider as shown in Table 1, and *ρ* is the total number of serviced cases. As seen in Figure 5, *α*_updated_ is calculated where *β*_accuracy_ represents the number of patient cases diagnosed accurately by the provider and *β*_total_ represents the overall number of patient cases diagnosed by the provider.

**Table 1:** Breast cancer diagnosis methods (providers).

Provider #	Method	Accuracy, %
1	CfS^a^ + SVM^b^ [35]	87.84
2	Filtered + SVM [35]	87.84
3	CfS + logistic regression [35]	95.95
4	Filtered + logistic regression [35]	96.62
5	BPSO^c^-2Stage [36]	92.98
6	PSO^d^ (4-2) [36]	93.98
7	KP^e^-SVM [37]	97.55
8	RFE^f^-SVM [37]	95.25
9	FSV^g^ [37]	95.23
10	Fisher + SVM [38]	94.70
11	Self-training [38]	85.12
12	Random co-training [38]	83.54
13	Rough co-training [38]	88.63
14	LDA^h^ [39]	97.19
15	C4.5 [39]	94.06
16	DIMLP^i^ [39]	96.92
17	SIM^j^ [39]	98.26
18	MLP^k^ [39]	97.43
19	PSO-KDE^l^ (1) [40]	98.45
20	PSO-KDE (2) [40]	98.45
21	GA^m^-KDE (2) [40]	98.45
22	Fisher + PFree Bat^n^ + LS^o^-SVM [41]	100

^a^CfS: correlation-based feature selection.

^b^SVM: support vector machine.

^c^BPSO: binary particle swarm optimization.

^d^PSO: particle swarm optimization.

^e^KP: kernel-penalized SVM (KP-SVM).

^f^RFE: recursive feature elimination.

^g^FSV: feature selection concave.

^h^LDA: linear discriminant analysis.

^i^DIMLP: discretized interpretable multilayer perceptron.

^j^SIM: similarity classifier.

^k^MLP: multilayer perceptron.

^l^KDE: kernel density estimation.

^m^GA: genetic algorithm.

^n^PFree Bat: parameter-free bat optimization algorithm.

^o^LS: least square support vector machine.

### Modeling the Service Requests

In the proposed system, everything is virtualized as a service according to the consensus standards. In this simulation, a computing service for breast tumor classification is completed. A Poisson random function is employed to simulate an interarrival time of generated service requests. The average interarrival time of service request is 3 minutes. A total of 9893 job requests are generated during 500 hours of the simulation run. Each service request includes a dataset containing a varying number of images to be analyzed. User priorities in terms of cost sensitivity and diagnostic accuracy sensitivity are reflected by the k-nearest neighbors (KNN) weights. Therefore, different users may have different KNN weight combinations.

### Design and Implementation of Breast Tumor Diagnosis Services

The price quote (*p*_quote_) for the job is calculated by *p*_quote_= *β* * *p*_unit_, in which *β* is the number of patient cases in the job and *p*_unit_ is calculated by *p*_unit_= *δ* + *ε* * *f* (*α*).

**Figure 6:**
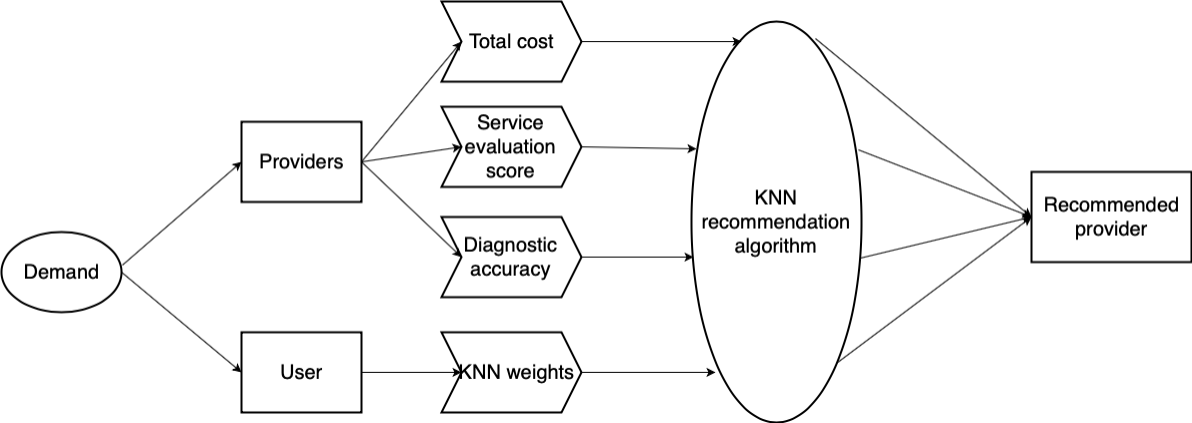
Process flow to obtain recommended provider. KNN: k-nearest neighbors.

Figure 6 shows the recommendation process based on the weighted KNN algorithm [42]. In this case, the total cost, diagnostic accuracy, and score of service evaluation are considered as the three nearest neighbors in the KNN algorithm. To estimate the different preferences of users, users have specific KNN weights. Based on KNN weight characteristics, users can be classified into four types. Type 1 users are price-oriented, and the weight of the total price is equal to or greater than 0.5; type 2 users are accuracy-oriented, and the weight of diagnostic accuracy is equal to or greater than 0.5; type 3 users are reputation-oriented, and the weight of the service evaluation score is equal to or greater than 0.5; and type 4 users are the normal users who do not have a single weight equal to or greater than 0.5.

**Figure 7:**
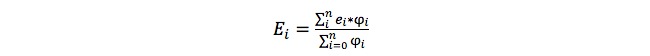
Equation of computing the service evaluation score.

**Figure 8:**
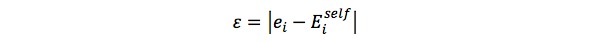
Equation of computing the difference between the user evaluation and the provider self-appraisal.

**Figure 9:**
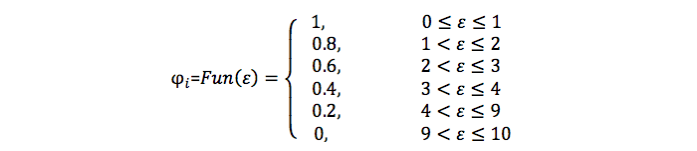
Equation of computing the probability of an arbitration request.

Peterson et al [43] indicate that the majority of conceptual distributions of satisfaction measurements follow a skewed distribution. As a result, a skewed distribution function is employed to model user evaluation results. Providers who have not successfully bid for a job will receive the average system service evaluation score. The equation in Figure 7 is used to compute the score of service evaluation of providers, where n represents the number of jobs that have been completed by the provider, e represents the evaluation score e∈[0,10], and *φ*_i_ is the evaluation coefficient of user, *φ*_i_∈{0,0.2,0.4,0.6,0.8,1}. The symbol *ε* represents the difference between the user evaluation and the provider self-appraisal, which is evaluated by the equation in Figure 8, in which *E*_i_^self^ represents the provider self-evaluation regarding the service. Finally, the value of *φ*_i_ is computed by the equation in Figure 9.

**Figure 10:**
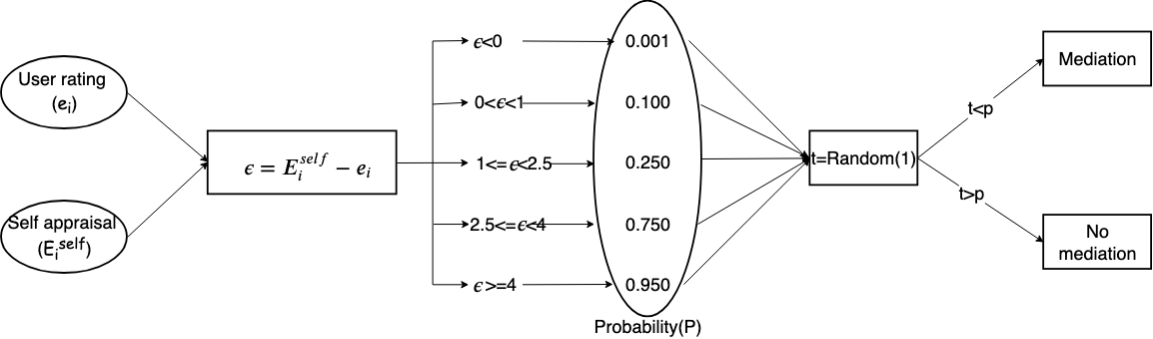
Process of determining arbitration for user ratings of service.

To simulate the process of arbitration, the difference between the provider self-appraisal and the user rating is calculated. The values of the difference are classified into several categories according to the probabilities as shown in Figure 10. A uniform distribution random value is generated to compare the values of probability. If the random value is less than the probability, arbitration is triggered, and vice versa.

## Results

**Figure 11:**
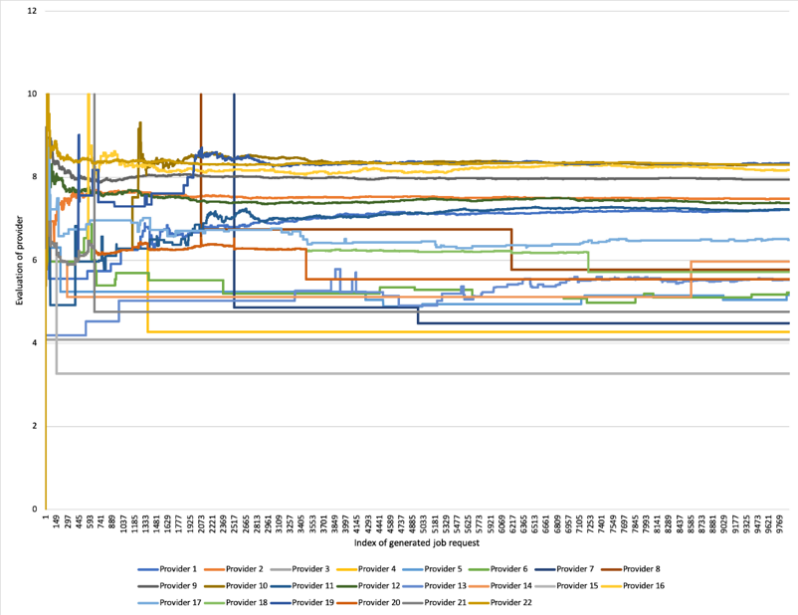
Service evaluation changes during simulation.

In the simulation, 500 hours of interarrival time of service request generation is simulated and 9893 service requests are generated. It is found that 15 providers have fulfilled all 9893 requests, while the other 7 providers have failed to take up any job. Figure 11 shows the appraisal of provider service given by users. The figure indicates that service evaluation scores of each provider stabilize with the increase of job index. This is because the service evaluation of users is related to the diagnostic accuracy, listed in Table 1. The figure shows rapid increases in evaluation score in the initial phase. This is because the service level scores of providers are set to 0 at the beginning, with scores updated after the providers start to fulfill service requests. However, for providers who fail to fulfill any job, changes in service evaluation score can also be observed. The reason is that those providers are assigned the average service evaluation score of the system. This mechanism enables all providers to have a winning opportunity to compete for jobs. It can also be found that the curves possess two different shapes. Some curves appear to comprise several major completely flat line segments, while others show a wavy pattern. The first type of curve shape implies that a provider only takes up a very limited number of service request. Many providers, such as providers 7, 8, and 21, show a sudden drop right after a sudden increase around service request numbers 2400, 2073, and 600, respectively. Those providers fulfill no service requests before that. Once they take up their first service requests, their service evaluation changes from the system average to a very high evaluation score. However, after that, those providers fulfill approximately 10 service requests in a short amount of time and then the service score reduces and stabilizes.

**Figure 12:**
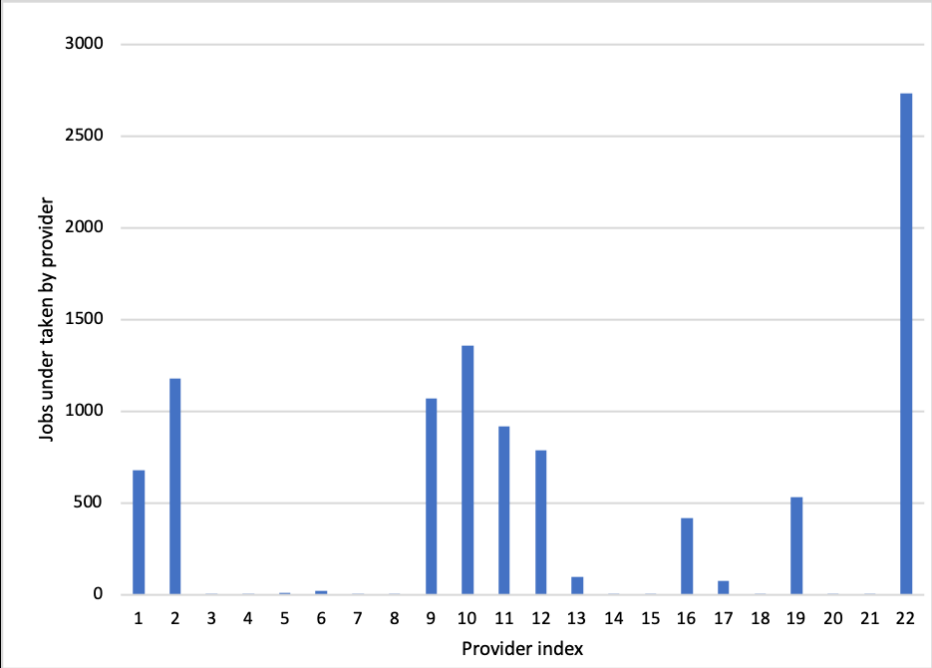
Number of service requests fulfilled by providers.

**Figure 13:**
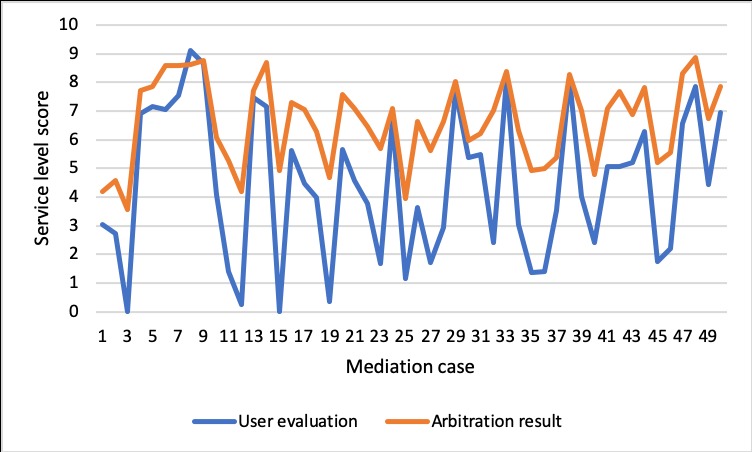
Evaluation of provider in first the 50 mediation cases.

Figure 12 illustrates the final total number of service requests fulfilled by each provider during the simulated 500 hours. It can be seen that provider 22 fulfills the most service requests, which comprise almost a quarter of the total requests. Providers 2, 9, and 10 fulfill more than 1000 service requests. Also, providers 1, 11, 12, and 19 represent the third echelon, with fulfilled requests higher than 500 but less than 1000. The 8 providers undertake 9254 out of 9853 service requests (more than 90%). Figure 13 shows the service score for the first 50 arbitration cases. It shows that the majority of mediation results are higher than the original user evaluations. The result implies that to a certain extent, the arbitration mechanism has the ability to fix the difference between the score given by the users and the actual service level of the provider. On the other hand, the arbitration results of cases 9, 13, 24, 29, 33, and 38 are very similar to the corresponding user evaluations. Moreover, it is evident that for case 8, the mediation result is actually lower than the user evaluation. The observations indicate that while the arbitration mechanism does offer the capability of fixing malicious reviews of the providers, it does not guarantee a better result than the user evaluation.

**Figure 14:**
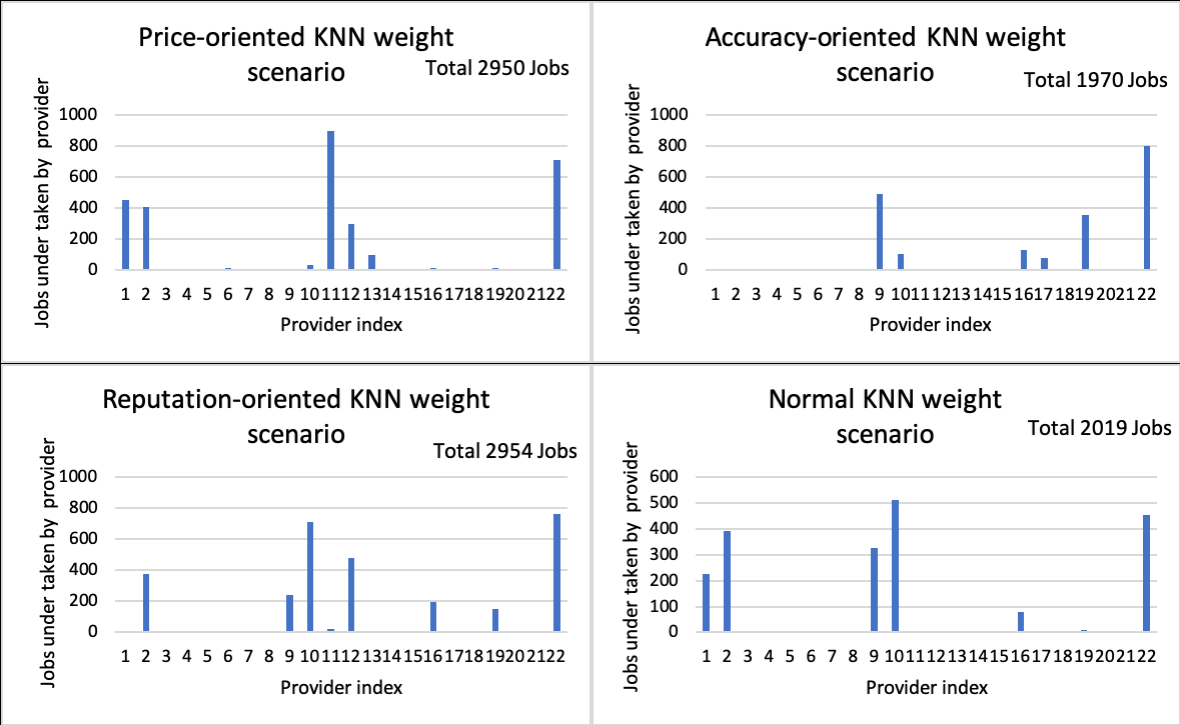
KNN weight sensitivity on provider selection. KNN: k-nearest neighbors.

Figure 14 shows the number of service requests each provider fulfills under each KNN weight scenario. The diagram illustrates that provider 1 fulfills around 450 service requests under the price-oriented KNN weight scenario compared with 0 under the other two KNN weight scenarios. Under the price-oriented KNN weight scenario, Provider 11 fulfills approximately 900 jobs, which is the highest compared with other providers. Provider 22, who provides the most accurate service, fulfills the highest number of service requests under the accuracy-oriented and reputation-oriented KNN weight scenarios, while fulfilling the second highest number of service requests under the price-oriented and normal KNN weight scenarios. This indicates that provider 22 is preferred over all other providers overall. Provider 2 fulfills a significant number of service requests under the price-oriented, reputation-oriented, and normal KNN weight scenarios but fulfills a negligible number of requests under the accuracy-oriented KNN weight scenario. This is because provider 2 has advantages in price and service but is less satisfactory in accuracy. Providers 1 and 11 fulfill more service requests under the price-oriented KNN weight scenario than they fulfill under the other three scenarios. The implication is that these two providers offer a reasonable price for service, but they have poor performance in user service ratings and diagnostic accuracy. Providers 9 and 19 are not effective under the price-oriented KNN weight scenario, but they are effective in the other three KNN scenarios indicating they adopted an unsuccessful strategy in price competition. These observations reflect the nature of the system design: providers may have a disadvantage in one aspect, but they can still win the competition by offering attractive conditions in other aspects.

## Discussion

In this research, we propose a model of cloud health service sharing-based blockchain technology featuring resource sharing, consensus, global payment, and distributed ledger. This mechanism allows the proposed framework to have sufficient feasibility and be supported by an increasing number of participants. Based on the open source Ethereum blockchain system, we adopt a consortium or federated blockchain for the proposed framework. Solidity language is employed to develop smart contracts. A simulation study on breast cancer diagnosis is constructed. A recommendation algorithm is designed to find the proper providers for service requests. During the 500 hour simulated time of generated service requests, 9893 job requests are generated and fulfilled by 22 providers. All requests are fulfilled by service providers based on recommendations from the weighted KNN algorithm, and 9 providers take up the preponderance of service requests. User priority on KNN weights evidently affect system operation outcomes. Provider service evaluation scores stabilize as service requests increase during the simulation.

A service evaluation system is incorporated in which a novel arbitration mechanism is designed to address the issue of potential biased evaluations. Both the self-appraisal of the provider and the evaluation by the user are taken into account in the arbitration. This protects providers by mitigating negative evaluations of malicious users. Note that the arbitration process adopts a distributed decision model through voting to mediate conflict. Qualified arbitration committee members could be distributed worldwide. When arbitration is submitted, members of the arbitration committee are selected by the system randomly and the selected members constitute the arbitration committee only for the case. The system sends the summary information of the case to the committee members. The committee members vote based on the shreds of verified evidence. The system finally reaches a decision by a smart contract.

We feel that the proposed model has tremendous potential, and the current work represents only the first step by demonstrating its feasibility. Research extension is called for to better design and optimize the proposed model in the future. For instance, for the proposed model to be implemented in the real world, the design of a cryptocurrency system is of great importance. The issue has not been addressed in this study, and it is worth investigation. Also, with the introduction of blockchain technology in the proposed model, some security issues could be mitigated but new challenges could arise, such as the high overhead of blockchain technology, privacy of the transaction, majority attack, the scale of blockchain, current regulations issues, and the integrated cost problem [44]. The new challenges should be dealt with in the future. In terms of business models on such a proposed system, interesting topics include pricing strategies for providers under a variety of situations. In addition, new applications of the proposed model should be explored addressing medicine prescription tracking and health insurance claims.

## Abbreviations

AI: artificial intelligence
CHC: cloud health coin
DApp: decentralized application
EHR: electronic health record
KNN: k-nearest neighbors
PoA: Proof of Authority
